# Vitamin C and Doxycycline: A synthetic lethal combination therapy targeting metabolic flexibility in cancer stem cells (CSCs)

**DOI:** 10.18632/oncotarget.18428

**Published:** 2017-06-09

**Authors:** Ernestina Marianna De Francesco, Gloria Bonuccelli, Marcello Maggiolini, Federica Sotgia, Michael P. Lisanti

**Affiliations:** ^1^ Department of Pharmacy, Health and Nutritional Sciences, University of Calabria, Rende, Italy; ^2^ The Paterson Institute, University of Manchester, Withington, United Kingdom; ^3^ Translational Medicine, School of Environment and Life Sciences, Biomedical Research Centre (BRC), University of Salford, Greater Manchester, United Kingdom

**Keywords:** cancer stem-like cells (CSCs), doxycycline, vitamin C, mitochondrial biogenesis, mitochondrial DNA (mt-DNA)

## Abstract

Here, we developed a new synthetic lethal strategy for further optimizing the eradication of cancer stem cells (CSCs). Briefly, we show that chronic treatment with the FDA-approved antibiotic Doxycycline effectively reduces cellular respiration, by targeting mitochondrial protein translation. The expression of four mitochondrial DNA encoded proteins (MT-ND3, MT-CO2, MT-ATP6 and MT-ATP8) is suppressed, by up to 35-fold. This high selection pressure metabolically synchronizes the surviving cancer cell sub-population towards a predominantly glycolytic phenotype, resulting in metabolic inflexibility. We directly validated this Doxycycline-induced glycolytic phenotype, by using metabolic flux analysis and label-free unbiased proteomics.

Next, we identified two natural products (Vitamin C and Berberine) and six clinically-approved drugs, for metabolically targeting the Doxycycline-resistant CSC population (Atovaquone, Irinotecan, Sorafenib, Niclosamide, Chloroquine, and Stiripentol). This new combination strategy allows for the more efficacious eradication of CSCs with Doxycycline, and provides a simple pragmatic solution to the possible development of Doxycycline-resistance in cancer cells. In summary, we propose the combined use of i) Doxycycline (Hit-1: targeting mitochondria) and ii) Vitamin C (Hit-2: targeting glycolysis), which represents a new synthetic-lethal metabolic strategy for eradicating CSCs.

This type of metabolic Achilles’ heel will allow us and others to more effectively “starve” the CSC population.

## INTRODUCTION

Cancer stem cells (CSCs) are thought to be the “root cause” of tumor recurrence, distant metastasis and therapy-resistance, driving poor clinical outcome in advanced cancer patients [1–3]. Therefore, new therapeutic strategies are necessary to identify and eradicate CSCs [4–7]. As such, this goal remains an unmet medical need.

Recently, we identified that high mitochondrial mass is a new common and characteristic feature of CSCs, based on high-resolution proteomics analysis [8–10]. Importantly, high mitochondrial mass is a surrogate marker for increased mitochondrial biogenesis and/or elevated mitochondrial protein translation. Thus, this simple metabolic observation provides a new means for both i) CSC identification [9–13] and ii) CSC eradication [9, 10, 14–19].

Specifically, we showed that a mitochondrial fluorescent dye (MitoTracker) could be effectively used for the enrichment and purification of CSC activity from a heterogeneous population of living cells [11–13]. In this context, cancer cells with the highest mitochondrial mass had the strongest functional ability to undergo anchorage-independent growth, a characteristic normally associated with metastatic potential [11–13]. The ‘Mito-high’ cell sub-population also had the highest tumor-initiating activity *in vivo*, as shown using pre-clinical models. High mitochondrial mass was strictly correlated with i) increased hTERT activity and ii) the ability to undergo cell proliferation, which was sensitive to CDK4/6 inhibitors, such as palbociclib [16]. Complementary results were obtained with other fluorescent mitochondrial probes for ROS and hydrogen peroxide, as well as NADH auto-fluorescence, an established marker of mitochondrial “power”/high OXPHOS activity [13].

Moreover, we demonstrated that several classes of non-toxic antibiotics could be used to halt CSC propagation [14–19]. Because of the conserved evolutionary similarities between aerobic bacteria and mitochondria, certain classes of antibiotics inhibit mitochondrial protein translation, as an off-target side-effect [14]. One such group of antibiotics is the tetracyclines, the prototypic family member being Doxycycline.

Through this analysis, it became apparent that tetracycline antibiotics, such as Doxycycline, could be re-purposed to eradicate CSCs, in multiple cancer types [14, 20, 21]. These eight distinct cancer types included: DCIS, breast (ER(+) and ER(−)), ovarian, prostate, lung, and pancreatic carcinomas, as well as melanoma and glioblastoma. Doxycycline was also effective in halting the propagation of primary cultures of CSCs from breast cancer patients, with advanced metastatic disease (isolated from ascites fluid and/or pleural effusions) [20].

Remarkably, Doxycycline behaves as a strong radio-sensitizer, successfully overcoming radio-resistance in breast CSCs [20]. This has important clinical implications, as the majority of ER(+) breast cancer patients are currently treated with breast-conserving surgery (lumpectomy) plus radiation therapy and hormonal therapy with an anti-estrogen.

Doxycycline is an FDA-approved drug, which first became available in 1967, ∼50 years ago now. It has excellent pharmacokinetic properties, with absorption of nearly 100% and a half-life of 18 to 24 hours. However, as with any new potential therapy, there is always a concern regarding the possible development of drug-resistance.

Here, we show that cancer cells can indeed escape the effects of Doxycycline, by reverting to a purely glycolytic phenotype. Fortunately, the metabolic inflexibility conferred by this escape mechanism allows Doxycycline-resistant (DoxyR) CSCs to be more effectively targeted with many other metabolic inhibitors, including Vitamin C, which functionally blocks aerobic glycolysis.

Interestingly, previous studies have shown that Vitamin C inhibits GAPDH (a glycolytic enzyme) and depletes the cellular pool of glutathione, resulting in high ROS production and oxidative stress [22]. We show here that DoxyR CSCs are between 4- to 10-fold more susceptible to the effects of Vitamin C, inhibiting their propagation in the range of 100 to 250 μM. Therefore, Doxycycline and Vitamin C may represent a new synthetic lethal drug combination for eradicating CSCs, by ultimately targeting both mitochondrial and glycolytic metabolism.

## RESULTS

Metabolic flexibility is the intrinsic ability of a cell to change from one carbon fuel source to another; conversely, metabolic inflexibility is the exact opposite: the lack of ability (or dramatically reduced ability) to change fuel sources. It is believed that metabolic flexibility in cancer cells allows them to escape therapeutic eradication, leading to chemo- and radio-resistance. Here, we used doxycycline to pharmacologically induce metabolic inflexibility in CSCs, by chronically inhibiting mitochondrial biogenesis. This treatment resulted in a purely glycolytic population of surviving cancer cells. Then, we identified six other clinically-approved therapeutics, two natural products and one experimental drug, that all successfully eradicate the remaining glycolytic CSCs. Therefore, Doxycycline-induced metabolic inflexibility may be a practical solution to avoiding treatment failure, in a variety of cancer types.

### Generating a Doxycycline-resistant MCF7 cell line, to study the potential mechanism(s) underlying drug resistance

**Figure 1:**
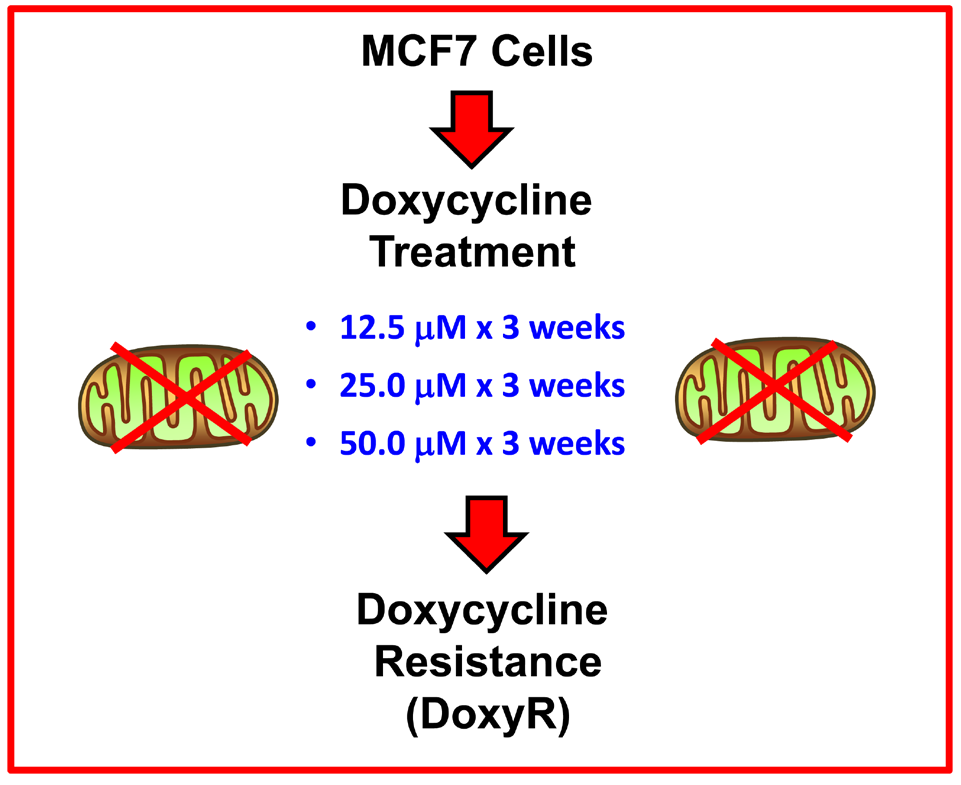
Generating MCF7 DoxyR cells Doxycycline-resistant (DoxyR) MCF7 cells were generated by serially passaging MCF7 cells, in the presence of increasing step-wise concentrations of Doxycycline (12.5, 25 and 50 μM), over a period of 9 weeks. See the *Materials and Methods* section for further details. Unless stated otherwise, MCF7 cells resistant to 25 μM Doxycycline were utilized for experiments, such as unbiased proteomics analysis.

To study the potential role of Doxycycline-resistance as an escape mechanism during Doxycycline treatment, we created a Doxycycline-resistant MCF7 cell line by serially passaging Doxycycline-sensitive MCF7 cells, in the presence of increasing concentrations of Doxycycline (from 12.5 to 50 μM), over a period of 9 weeks. The experimental procedure we utilized is briefly outlined in Figure 1 and is detailed in the *Materials and Methods* section.

**Figure 2:**
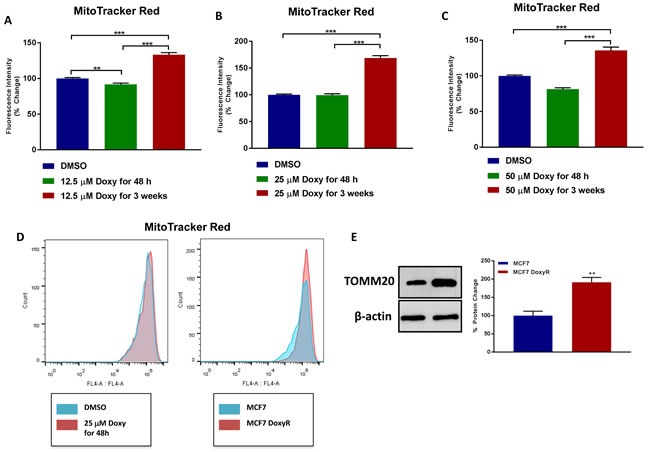
MCF7 DoxyR cells exhibit an increase in mitochondrial mass **A.**-**D.** MCF7 cells were treated with DMSO or Doxycycline for acute (48 h) and chronic stimulation (3 weeks), as specified in *Materials and Methods*, and then mitochondrial mass was quantitated by FACS analysis using the probe MitoTracker Deep-Red (640-nm). Note that MCF7 cells chronically treated with 12.5 μM (A., *fold change 1.33*), 25 μM (B., *fold change 1.68*) and 50 μM (C., *fold change 1.36*) Doxycycline show a significant increase in mitochondrial mass compared to MCF7 cells treated with vehicle. Data shown are the mean ± SEM of at least 3 independent experiments performed in triplicate. (**) *p* < 0.01; (***) *p* < 0.001. D. Representative plots showing increased mitochondrial mass in MCF7 DoxyR cells as compared to MCF7 cells. **E**. Evaluation of the mitochondrial protein TOMM20 in MCF7 and MCF7 DoxyR cells by western blotting. Side panel shows densitometric analysis of the blots normalized to β-actin. Data shown are the mean ± SEM of 3 independent experiments. (**) *p* < 0.01.

Doxycycline-treated MCF7 cells were analyzed at each stage for mitochondrial mass. As shown in Figure 2A-2D, Doxycycline-resistant (DoxyR) MCF7 cells show a significant increase in mitochondrial mass (by ∼1.3- to 1.7-fold), as compared to acute treatment with Doxycycline, at the same drug concentration. This overall increase in mitochondrial mass was confirmed by immuno-blot analysis with specific antibodies directed against TOMM20, a well-established marker of mitochondrial mass (Figure 2E).

**Figure 3:**
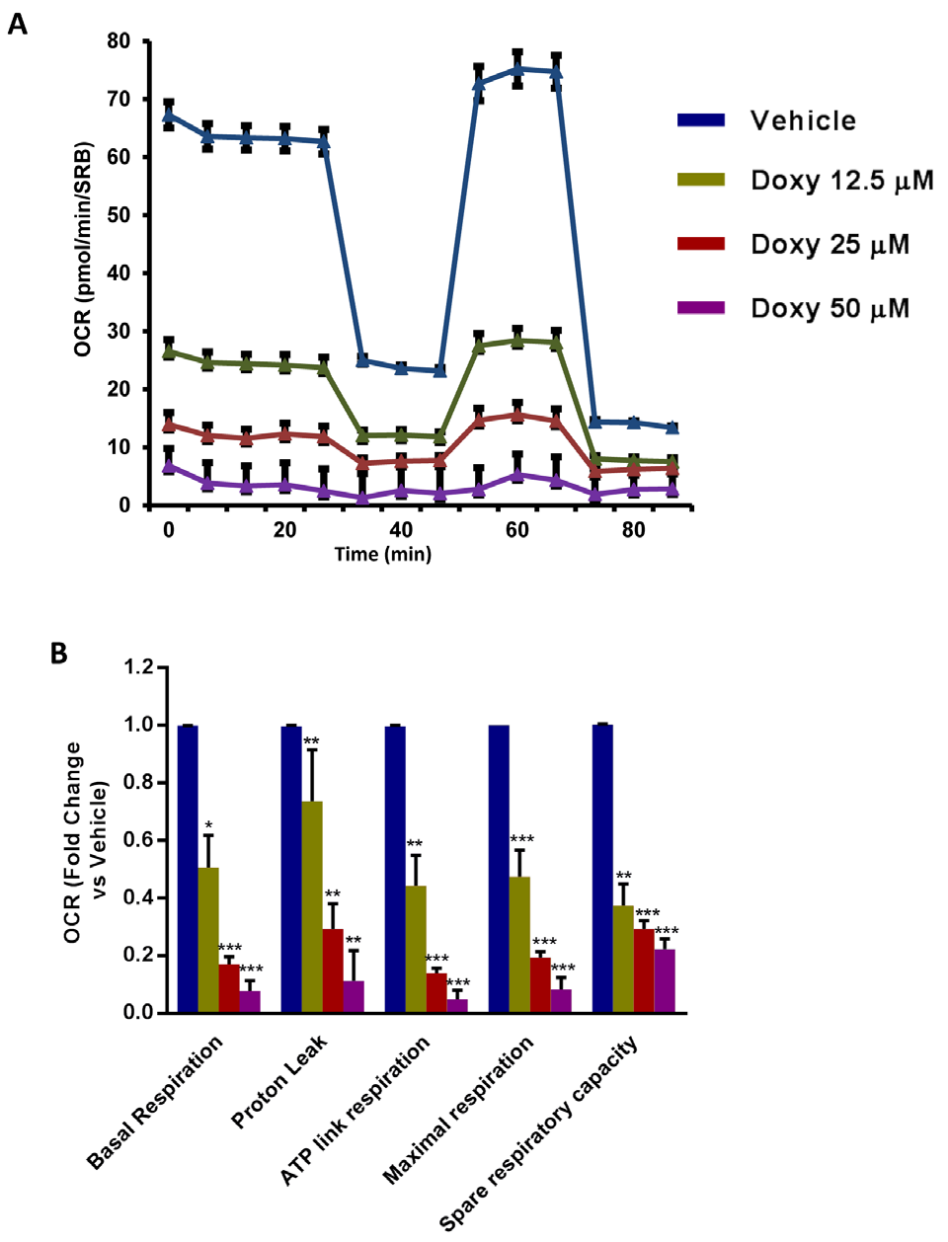
Mitochondrial respiration is inhibited in MCF7 DoxyR cells The metabolic profile of MCF7 DoxyR cells monolayers chronically treated with increasing concentrations of Doxycycline (12.5 μM ÷ 50 μM), as described in Materials and Methods, was assessed using the Seahorse XF-e96 analyzer. **A**. Representative tracing of metabolic flux. Dose-dependent significant reduction in basal respiration, proton leak, maximal respiration, ATP levels and spare respiratory capacity were observed **B**. Data shown are the mean ± SEM of 3 independent experiments performed in sextuplicate. (*) *p* < 0.05; (**) *p* < 0.01; (***) *p* < 0.001.

**Figure 4:**
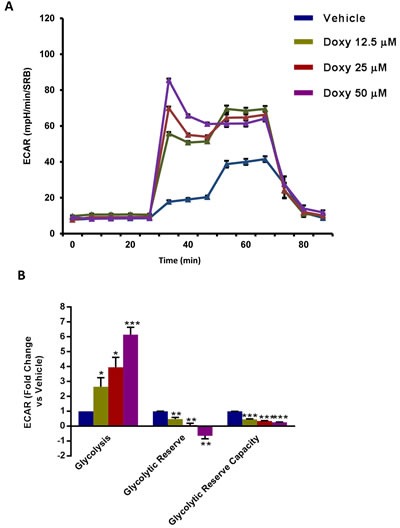
Glycolysis is increased in MCF7 DoxyR cells The metabolic profile of MCF7 DoxyR cells monolayers chronically treated with increasing concentrations of Doxycycline (12.5 μM ÷ 50 μM), as described in Materials and Methods, was assessed using the Seahorse XF-e96 analyzer. **A**. Representative tracing of metabolic flux. **B**. Dose-dependent significant increase in glycolysis and decrease in glycolytic reserve as well as glycolytic reserve capacity were observed. Data shown are the mean ± SEM of 3 independent experiments performed in sextuplicate. (*) *p* < 0.05; (**) *p* < 0.01; (***) *p* < 0.001.

To understand the effects of chronic Doxycycline treatment on cell metabolism, we next performed metabolic flux analysis with the Seahorse XFe96. Interestingly, Figure 3 illustrates that MCF7-DoxyR cells show a dramatic reduction in oxygen consumption rates (OCR), as compared to matched control MCF7 cells, processed in parallel. As a consequence, ATP levels were severely depleted. Conversely, glycolysis was substantially increased, as measured by the ECAR (extracellular acidification rate) (Figure 4). Therefore, DoxyR cells are mainly glycolytic. As such, a sub-population of MCF7 cells survive and develop Doxycycline-resistance, by adopting a purely glycolytic phenotype.

### Doxycycline-resistant MCF7 cells show an increase in CSC markers, but not in functional CSC activity, as measured using mammosphere assays, proliferation and cell migration

**Figure 5:**
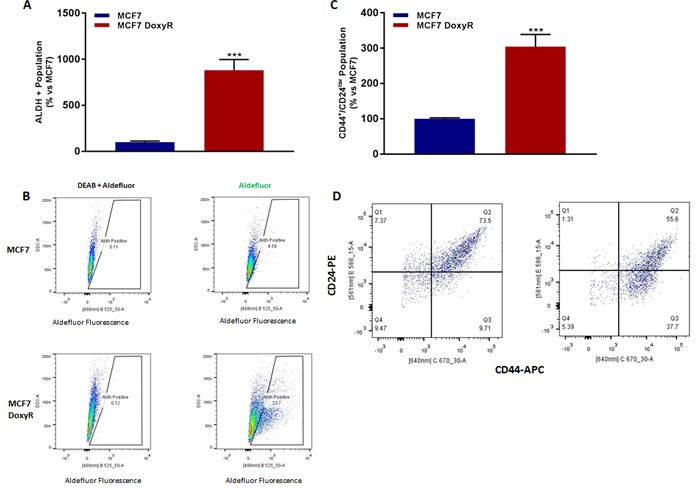
MCF7 DoxyR cells show increased CSC markers 48h after seeding, MCF7 and MCF7 DoxyR cells were processed for the evaluation of ALDEFLUOR activity, an independent marker of CSCs. Each sample was normalized using diethylaminobenzaldehyde (DEAB), a specific ALDH inhibitor, as negative control **A**. The tracing of representative samples is shown **B**. 48h after seeding, MCF7 and MCF7 DoxyR cells were re-plated on low-attachment plates, for anoikis assay for 10 hours. Expression of CSC markers (CD24 and CD44) was analysed by FACS **C**. Representative dot plot for the the CD44+/CD24low cell population is shown **D**. This represents an ∼10-fold increase in ALDH functional activity and a ∼3-fold induction of the CD44+/CD24low population. Data are the mean ± SEM of 3 independent experiments performed in triplicate. (***) *p* < 0.001.

**Figure 6:**
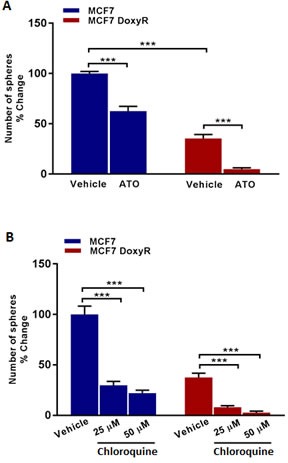
Mammosphere formation is inhibited in MCF7 DoxyR cells: Targeting DoxyR cells with Atovaquone and Chloroquine Evaluation of mammosphere formation in MCF7 and MCF7 DoxyR cells cultured in low attachment plates and treated with vehicle or the selective OXPHOS inhibitor Atovaquone (ATO) **A**. or Chloroquine **B**. (which has been shown to impair mitochondrial metabolism), for 5 days before counting. Note that sphere formation is inhibited in MCF7 DoxyR cells as compared to MCF7 cells. In addition, mitochondrial-targeting agents like atovaquone and Chloroquine were effective in reducing the number of spheres in both MCF7 and MCF7 DoxyR cells. Data shown are the mean ± SEM of 3 independent experiments performed in triplicate. (***) *p* < 0.001.

ALDH activity and CD44/CD24 levels are routinely used as typical markers to identify breast CSCs [1–7]. Interestingly, MCF7-DoxyR cells show a substantial increase in these two CSC markers, as revealed by FACS analysis (Figure 5). However, these markers do not reflect CSC activity. To more directly assess functional CSC activity, we used the mammosphere assay. Remarkably, MCF7-DoxyR cells show a > 60% reduction in CSC activity using the mammosphere assay as a readout (Figure 6). Therefore, the increases in CSC markers that we observed do not actually reflect a functional increase in CSC propagation.

**Figure 7:**
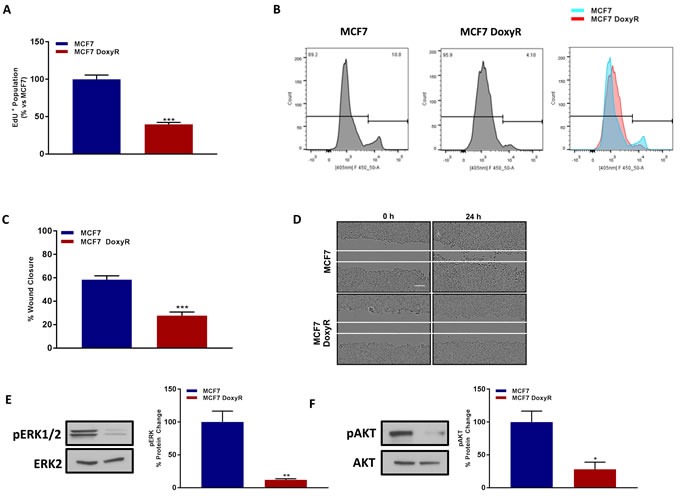
MCF7 DoxyR cells show a quiescent phenotype, with significantly reduced proliferation and cell migration, as well as suppression of ERK- and AKT-signaling Evaluation of cell proliferation by EdU incorporation assay using FACS analysis in MCF7 and MCF7 DoxyR cells 48h after seeding **A**. Note the reduction in EdU positive population in MCF7 DoxyR cells as compared to MCF7 cells. The tracing of a representative sample is shown **B**. Data shown are the mean ± SEM of 4 independent experiments performed in triplicate. (***) *p* < 0.001. Evaluation of cell migration by wound healing assay in MCF7 and MCF7 DoxyR cells which were seeded in 6 well plate to create a confluent monolayer. 24h after seeding a wound was created, then cells were washed and incubated at 37°C for 24 h. Images were acquired at 0 h and 24 h using Incucyte Zoom (Essen Bioscience). Quantification of cell migration was performed using ImageJ software and was expressed as % of wound closure **C**. Note the low migratory capacity of MCF7 DoxyR cells as compared to MCF7 cells. Representative images showing scratch assay **D**. Bar scale 100 μm. Data shown are the mean ± SEM of 3 independent experiments performed in triplicate. (***) *p* < 0.001. Evaluation of ERK1/2 **E**. and AKT Ser 473 **F**. phosphorylation in MCF7 and MCF7 DoxyR cells by western blotting. Side panels show densitometric analysis of the blots normalized to ERK2 and AKT respectively. Data shown are the mean ± SEM of 3 independent experiments. (*) *p* < 0.05; (**) *p* < 0.01.

Consistent with our above findings using the mammosphere assay, MCF7-DoxyR cells appear to be relatively quiescent, as they show dramatic reductions in their ability to proliferate by > 60%, as measured using EdU-incorporation, which reflects reduced DNA-synthesis (Figure 7A, 7B). Similarly, MCF7-DoxyR cells also show a clear defect in cell migration, with a > 50% reduction, as observed using the standard “scratch assay” (Figure 7C, 7D).

Taken together, these findings are consistent with an overall tendency towards a quiescent glycolytic cell phenotype. Consistent with this assertion, DoxyR cells also show dramatic reductions in ERK-activation and AKT-activation, as revealed by immuno-blot analysis, with phospho-specific antibody probes (Figure 7E, 7F).

### Proteomics analysis of MCF7-DoxyR cells provides validating evidence for a predominantly glycolytic phenotype, due to a loss of mitochondrial function

In order to further validate our functional observations from metabolic flux analysis, we also performed unbiased label-free proteomics analysis [8]. These results are summarized and presented in Tables 1–5. Based on this comprehensive proteomics analysis, MCF7-DoxyR cells show severe reductions in mitochondrial proteins, both those encoded by mitochondrial DNA (mt-DNA) and those encoded by nuclear DNA (nuc-DNA).

**Table 1:** Key Mitochondrial-related Proteins are Down-regulated in Doxy-Resistant MCF7 Cells

Symbol	Description		Fold-reduction (Down-regulation)
**Mitochondrial proteins encoded by mitochondrial DNA**			
MT-ND3	NADH-ubiquinone oxidoreductase chain 3	(Complex I)	35.07
MT-CO2	Cytochrome c oxidase subunit 2	(Complex IV)	19.26
MT-ATP8	ATP synthase protein 8	(Complex V)	6.42
MT-ATP6	ATP synthase subunit 6	(Complex V)	5.08
			
**Mitochondrial proteins encoded by nuclear DNA**			
NDUFS1	NADH-ubiquinone oxidoreductase 75 kDa subunit, mitochondrial		12.53
NNT	NAD(P) transhydrogenase, mitochondrial		10.49
SSBP1	Single-stranded DNA-binding protein, mitochondrial		9.27
NDUFB8	NADH dehydrogenase 1 beta subcomplex subunit 8, mitochondrial		8.50
CKMT1A	Creatine kinase U-type, mitochondrial		7.49
TFAM	Transcription factor A, mitochondrial		6.89
COX7C	Cytochrome c oxidase subunit 7C, mitochondrial		5.40
COX7A2	Cytochrome c oxidase subunit 7A2, mitochondrial		5.34
SDHB	Succinate dehydrogenase iron-sulfur subunit, mitochondrial		4.86
COX5B	Cytochrome c oxidase subunit 5B, mitochondrial		4.83
CKMT2	Creatine kinase S-type, mitochondrial		4.78
COQ6	Ubiquinone biosynthesis monooxygenase COQ6, mitochondrial		4.71
HYOU1	Hypoxia up-regulated protein 1		4.55
CHDH	Choline dehydrogenase, mitochondrial		4.42
NDUFV1	NADH dehydrogenase [ubiquinone] flavoprotein 1, mitochondrial		4.31
PUS1	tRNA pseudouridine synthase A, mitochondrial		4.28
OXCT1	Succinyl-CoA:3-ketoacid coenzyme A transferase 1, mitochondrial		4.17
TOMM6	Mitochondrial import receptor subunit TOM6		4.15
ACAA2	3-ketoacyl-CoA thiolase, mitochondrial		4.04
NFU1	NFU1 iron-sulfur cluster scaffold homolog, mitochondrial		3.96
CPT1A	Carnitine O-palmitoyltransferase 1, liver isoform		3.52
UQCRC1	Cytochrome b-c1 complex subunit 1, mitochondrial		3.51
PRKDC	DNA-dependent protein kinase catalytic subunit		3.43
MDH2	Malate dehydrogenase, mitochondrial		3.30
ACSF3	Acyl-CoA synthetase family member 3, mitochondrial		3.29
FH	Fumarate hydratase, mitochondrial		3.27
PDHX	Pyruvate dehydrogenase protein X component, mitochondrial		3.23
BDH1	D-beta-hydroxybutyrate dehydrogenase, mitochondrial		3.16
NDUFS3	NADH dehydrogenase iron-sulfur protein 3, mitochondrial		3.16
MMAB	Cob(I)yrinic acid a,c-diamide adenosyltransferase, mitochondrial		3.12
DARS2	Aspartate--tRNA ligase, mitochondrial		3.00
SUCLA2	Succinyl-CoA ligase [ADP-forming] subunit beta, mitochondrial		2.91
ABAT	4-aminobutyrate aminotransferase, mitochondrial		2.83
LACTB	Serine beta-lactamase-like protein LACTB, mitochondrial		2.81
CHDH	Choline dehydrogenase, mitochondrial		2.78
GLS	Glutaminase kidney isoform, mitochondrial		2.77
TOMM34	Mitochondrial import receptor subunit TOM34		2.76
NDUFA10	NADH dehydrogenase 1 alpha subcomplex subunit 10, mitochondrial		2.70
MUL1	Mitochondrial ubiquitin ligase activator of NFKB 1		2.60
UQCRC2	Cytochrome b-c1 complex subunit 2, mitochondrial		2.54
COX7A2L	Cytochrome c oxidase subunit 7A-related protein, mitochondrial		2.54
SLC25A24	Calcium-binding mitochondrial carrier protein SCaMC-1		2.51
NDUFA9	NADH dehydrogenase 1 alpha subcomplex subunit 9, mitochondrial		2.50
GLUL	Glutamine synthetase		2.50
PDHA1	Pyruvate dehydrogenase E1 subunit alpha, somatic, mitochondrial		2.50
SDHA	Succinate dehydrogenase flavoprotein subunit, mitochondrial		2.48
NDUFS8	NADH dehydrogenase iron-sulfur protein 8, mitochondrial		2.42

**Table 2:** Enzymes Related to Glycolysis and Glycogen Metabolism are Up-regulated in Doxy-Resistant MCF7 Cells

Symbol	Description	Fold-Increase (Up-regulation)
**Glycolytic enzymes**		
PGM1	Phosphoglucomutase-1	7.16
LDHA	L-lactate dehydrogenase A	7.09
ALDOC	Fructose-bisphosphate aldolase C	3.44
GAPDH	Glyceraldehyde-3-phosphate dehydrogenase	3.06
GPD1L	Glycerol-3-phosphate dehydrogenase 1-like protein	2.72
ALDOA	Fructose-bisphosphate aldolase A	2.71
PFKP	ATP-dependent 6-phosphofructokinase, platelet type	2.69
PGK1	Phosphoglycerate kinase 1	2.64
GPI	Glucose-6-phosphate isomerase	2.46
PKM	Pyruvate kinase	2.10
		
**Glycogen metabolism**		
GYS1	Glycogen [starch] synthase, muscle	4.11
PYGM	Glycogen phosphorylase, muscle form	3.45
PYGL	Glycogen phosphorylase, liver form	3.39

**Table 3:** Markers of Hypoxia and Cancer Stem Cells are Up-regulated in Doxy-Resistant MCF7 Cells

Symbol	Description	Fold-Increase (Up-regulation)
**Hypoxia markers**		
MB	Myoglobin	5.86
HBA1	Hemoglobin subunit alpha	3.46
HBD	Hemoglobin subunit delta	1.81
		
**ALDH gene isoforms**		
ALDH1A3	Aldehyde dehydrogenase family 1 member A3	1,681.32
ALDH1A2	Retinal dehydrogenase 2	5.22
ALDH5A1	Succinate-semialdehyde dehydrogenase, mitochondrial	3.87
ALDH18A1	Delta-1-pyrroline-5-carboxylate synthase	2.75
ALDH16A1	Aldehyde dehydrogenase family 16 member A1	2.04
		
**Other cancer stem cell (CSC) markers**		
RGAP2	SLIT-ROBO Rho GTPase-activating protein 2	2.80
CD44	CD44 antigen	2.09

**Table 4:** A Subset of Mitochondrial Ribosomal Proteins (MRPs) are Increased in Doxy-Resistant MCF7 Cells

Symbol	Description	Fold-Increase (Up-regulation)
**Small subunit**		
MRPS25	28S ribosomal protein S25, mitochondrial	3.02
MRPS9	28S ribosomal protein S9, mitochondrial	1.69
MRPS18C	28S ribosomal protein S18c, mitochondrial	1.58
**Large subunit**		
MRPL10	39S ribosomal protein L10, mitochondrial	2.90
MRPL12	39S ribosomal protein L12, mitochondrial	2.21
MRPL46	39S ribosomal protein L46, mitochondrial	2.13
MRPL53	39S ribosomal protein L53, mitochondrial	2.13
MRPL37	39S ribosomal protein L37, mitochondrial	2.05
MRPL19	39S ribosomal protein L19, mitochondrial	1.95
MRPL15	39S ribosomal protein L15, mitochondrial	1.94

**Table 5:** A Subset of Cellular Ribosomal Proteins are Decreased in Doxy-Resistant MCF7 Cells

Symbol	Description	Fold-reduction (Down-regulation)
**Small subunit**		
RPS15	40S ribosomal protein S15	2.12
RPS21	40S ribosomal protein S21	2.08
RPS4X	40S ribosomal protein S4, X isoform	2.06
RPS23	40S ribosomal protein S23	1.82
**Large subunit**		
RPL34	60S ribosomal protein L34	9.85
RPL3	60S ribosomal protein L3	6.39
RPLP2	60S acidic ribosomal protein P2	3.68
RPL10A	60S ribosomal protein L10a	2.28
RPL27A	60S ribosomal protein L27a	2.06
RPL8	60S ribosomal protein L8	1.93
RPL22L1	60S ribosomal protein L22-like 1	1.82
**Other**		
RSL1D1	Ribosomal L1 domain-containing protein 1	3.08

A loss of mt-DNA-encoded proteins is characteristic hallmark of the inhibition of mitochondrial protein translation. Therefore, MCF7-DoxyR cells should be expected to metabolically phenocopy a mt-DNA-deficient genotype (rho(0) cells). For example, the cellular levels of MT-ND3, MT-CO2, MT-ATP6 and MT-ATP8 are all reduced between 5- to 35-fold (Table 1), which should inactivate or impair Complex I, IV and V. Similarly, > 45 nuclear-encoded mitochondrial proteins, such as NDUFS1, NDUFB8 and COX7C, are all decreased between 2- and 12-fold (Table 1).

In striking contrast, the levels of 10 glycolytic enzymes were all increased between 2- and 7-fold, including PGM1, LDHA, ALDOC and GAPDH (Table 2). Similarly, enzymes associated with glycogen metabolism were also increased, between 3- and 4-fold (GYS1, PYGM, PYGL); markers of hypoxia were also elevated (myoglobin and hemoglobin (alpha/delta)) (Table 3), supporting a predominant glycolytic phenotype. Consistent with an increase in Aldefluor activity, several ALDH gene products were increased, especially ALDH1A3. This increased ALDH activity may reflect their tendency towards glycolysis, as ALDH isoforms contribute significantly to the glycolytic pathway.

Table 4 shows that 10 mitochondrial ribosomal proteins (MRPs) were increased, between 1.5- to 3-fold. This would mechanistically explain the compensatory increase in mitochondrial mass observed in Figure 2.

Finally, Table 5 illustrates that 12 cellular ribosomal proteins were clearly down-regulated, between 1.8- and 9-fold, which may drive a severe decrease in cellular protein synthesis, due to mitochondrial energy deficits, resulting in a relatively quiescent metabolic phenotype.

### A synthetic lethal strategy for eradicating DoxyR CSCs, using atovaquone or chloroquine

Thus far, our experimental results indicate that DoxyR cells acquire a predominantly glycolytic phenotype, to escape the anti-mitochondrial effects of Doxycycline. This means that DoxyR cells have been inadvertently metabolically synchronized and suffer from a type of functional metabolic inflexibility. As such, they should be extremely sensitive to additional metabolic stressors or perturbations, allowing them to be eliminated completely. This immediately suggests a new synthetic lethal strategy for the metabolic eradication of CSCs, to avoid any resistance to Doxycycline.

**Figure 8:**
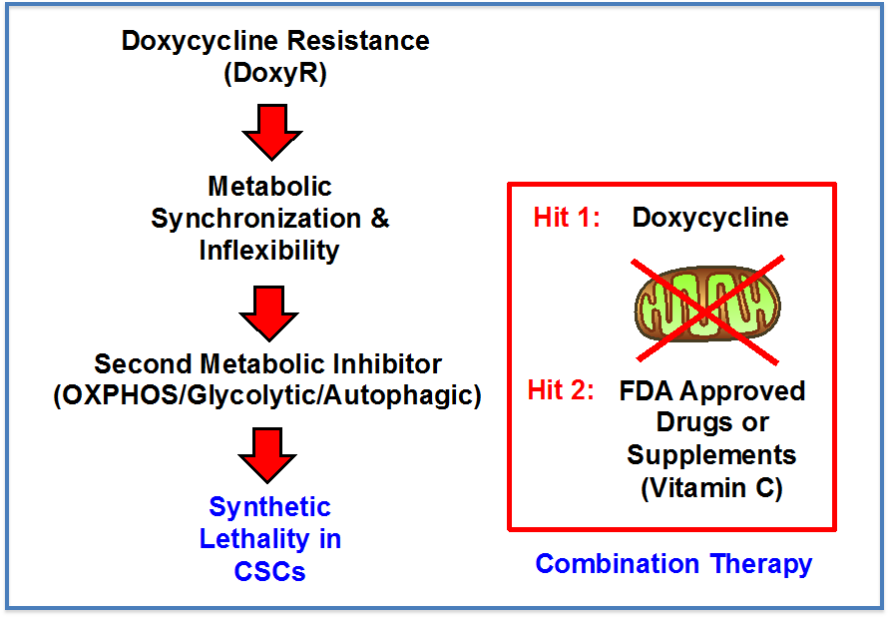
A two-hit synthetic lethal strategy for eradicating DoxyR CSCs Here, we outline a new therapeutic strategy for targeting CSCs. Our experimental results indicate that DoxyR cells acquire a predominantly glycolytic phenotype, to escape the anti-mitochondrial effects of Doxycycline. As such, they should be extremely sensitive to additional metabolic stressors, allowing them to be eliminated completely. This immediately suggests a new synthetic lethal strategy for the metabolic eradication of CSCs, to avoid resistance to Doxycycline. Specifically, if we consider DoxyR as the first metabolic Hit in a two-Hit scheme, then DoxyR cells should be extremely susceptible to a second metabolic Hit. This second metabolic Hit could be achieved by using virtually any other “safe” metabolic inhibitors, targeting either glycolysis, OXPHOS or autophagy.

More specifically, if we consider DoxyR as the first metabolic Hit in a two-Hit scheme, then DoxyR cells should be extremely susceptible to a second metabolic Hit. This second metabolic Hit could be achieved by using virtually any other “safe” metabolic inhibitors, targeting either glycolysis, OXPHOS or autophagy. This two-Hit metabolic scheme is illustrated schematically in Figure 8.

**Figure 9:**
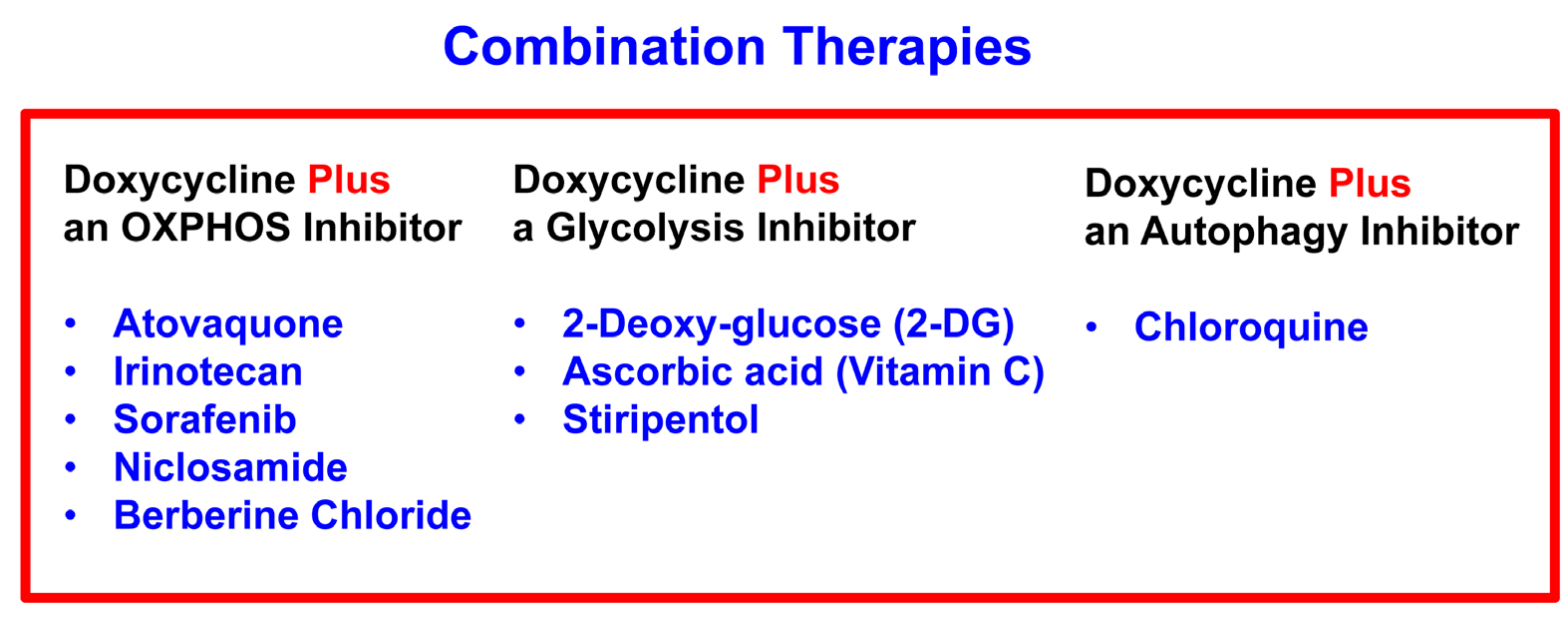
Metabolic inhibitors successfully employed for the eradication of DoxyR CSCs Briefly, a list of small molecules that we successfully used in conjunction with Doxycycline is shown. These include 9 known inhibitors of OXPHOS, glycolysis and autophagy. Two natural products (Vitamin C and Berberine), six clinically-approved drugs (Atovaquone, Chloroquine, Irinotecan, Sorafenib, Niclosamide, and Stiripentol) and one experimental drug (2-DG), are all highlighted.

To test this hypothesis, we first used Atovaquone, an FDA-approved OXPHOS inhibitor, which targets mitochondrial Complex III. Similarly, we examined the effects of Chloroquine, a well-known autophagy inhibitor [17]. Both Atovaquone and Chloroquine are normally used clinically for the treatment and prevention of malaria, a parasitic infection. A list summarizing these metabolic inhibitors is presented in Figure 9.

Importantly, Figure 6 shows that DoxyR CSC propagation is clearly more sensitive to Atovaquone, as compared with control MCF7 CSCs. More specifically, treatment with Atovaquone (1 μM) inhibited the CSC propagation of the DoxyR cells by > 85%. Previously, we showed that the IC-50 for Atovaquone was 1 μM for MCF7 CSC propagation [17]. Similarly, Chloroquine inhibited their propagation by > 75% at 25 μM and by > 90% at 50 μM. Thus, it is possible to target the propagation of DoxyR CSCs, using existing FDA-approved OXPHOS and autophagy inhibitors.

### A synthetic lethal strategy for eradicating DoxyR CSCs, using natural products (Vitamin C and Berberine) and other FDA-approved drugs

**Figure 10:**
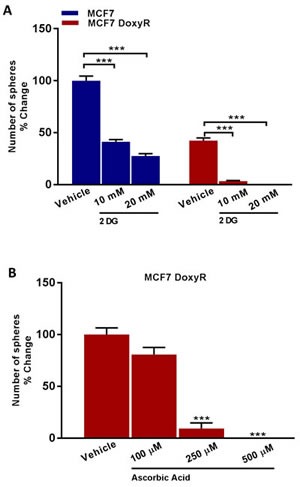
Glycolysis inhibitors reduce mammosphere formation in MCF7 DoxyR cells Evaluation of mammosphere formation in MCF7 and MCF7 DoxyR cells cultured in low attachment plates and treated with Vehicle or increasing concentrations of the glycoysis inhibitor 2-deoxy-glucose (2 DG) (10 mM to 20 mM) for 5 days before counting **A**. Mammosphere formation is inhibited in MCF7 DoxyR cells cultured in low attachment plates and treated with increasing concentrations of the glycoysis inhibitor Ascorbic Acid (100 μM to 500 μM) for 5 days before counting **B**. Data shown are the mean ± SEM of 3 independent experiments performed in triplicate. (***) *p* < 0.001.

Next, we tested the efficacy of glycolysis inhibitors, such as 2-deoxy-glucose (2-DG) and Vitamin C (ascorbic acid). Treatment with 2-DG inhibited the propagation of DoxyR CSCs by > 90% at 10 mM and 100% at 20 mM (Figure 10). In addition, Vitamin C was more potent than 2-DG; it inhibited DoxyR CSC propagation by > 90% at 250 μM and 100% at 500 μM (Figure 10). As such, the IC-50 for Vitamin C in this context was between 100 to 250 μM, which are within the known achievable blood levels, when Vitamin C is taken orally. Previously, we showed that the IC-50 for Vitamin C was 1 mM for MCF7 CSC propagation [13]. Therefore, DoxyR CSCs are between 4- to 10-fold more sensitive to Vitamin C than control MCF7 CSCs, under identical assay conditions.

**Figure 11:**
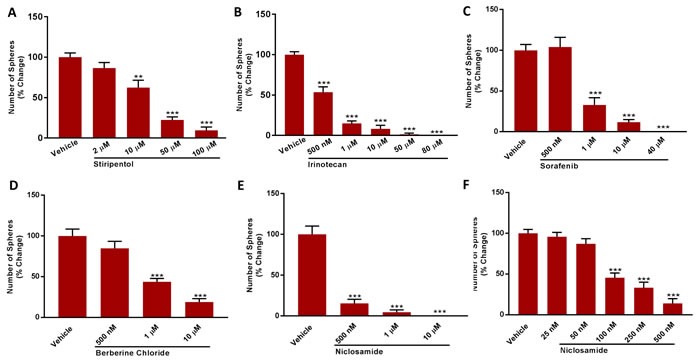
A panel of clinically-approved drugs inhibits mammosphere formation in MCF7 DoxyR cells Evaluation of mammosphere formation in MCF7 DoxyR cells cultured in low attachment plates and treated with Vehicle or increasing concentrations of the LDH inhibitor Stiripentol (2 μM to 100 μM) **A**. or the OXPHOS inhibitors Irinotecan (500 nM to 80 μM) **B**., Sorafenib (500 nM to 40 μM) **C**., Berberine Chloride (500 nM to 10 μM) **D**. and Niclosamide **E**.-**F**. for 5 days before counting. Data shown are the mean ± SEM of 3 independent experiments performed in triplicate. (**) *p* < 0.01: (***) *p* < 0.001.

We also examined the efficacy of 4 other clinically-approved drugs that functionally behave as either OXPHOS inhibitors (Irinotecan, Sorafenib, Niclosamide) or glycolysis inhibitors (Stiripentol) (Figure 11). In this context, Stiripentol functions as an LDH inhibitor. Their rank order potency for inhibiting DoxyR CSC propagation is: Niclosamide (IC-50 ∼ 100 nM) > Irinotecan (IC-50 ∼ 500 nM) > Sorafenib (IC-50 ∼ 0.5 to 1 μM) > Stiripentol (IC-50 ∼ 10 to 50 μM).

Finally, we tested the efficacy of Berberine, which is a naturally occurring antibiotic that also behaves as an OXPHOS inhibitor. It was used as early as 3,000 B.C. in China, for medicinal purposes. Figure 11 shows that treatment with Berberine effectively inhibited the propagation of the DoxyR CSCs by > 50% at 1 μM and > 80% at 10 μM.

## DISCUSSION

In this report, we present new functional evidence to support a novel synthetic lethal strategy to eradicate CSCs. More specifically, we demonstrate that the use of Doxycycline, a clinically approved antibiotic, induces metabolic stress in cancer cells. This allows the remaining cancer cells to be synchronized towards a purely glycolytic phenotype, driving a form of metabolic inflexibility. This Doxycycline-driven aerobic glycolysis was further confirmed and validated, by employing high-resolution proteomics analysis and metabolic phenotyping. In addition, we discovered that both natural products and FDA-approved drugs could be re-purposed to eradicate the Doxycycline-resistant CSC population. These 9 small molecules included: Vitamin C, Berberine, 2-DG, Atovaquone, Irinotecan, Sorafenib, Niclosamide, Chloroquine, and Stiripentol.

**Figure 12:**
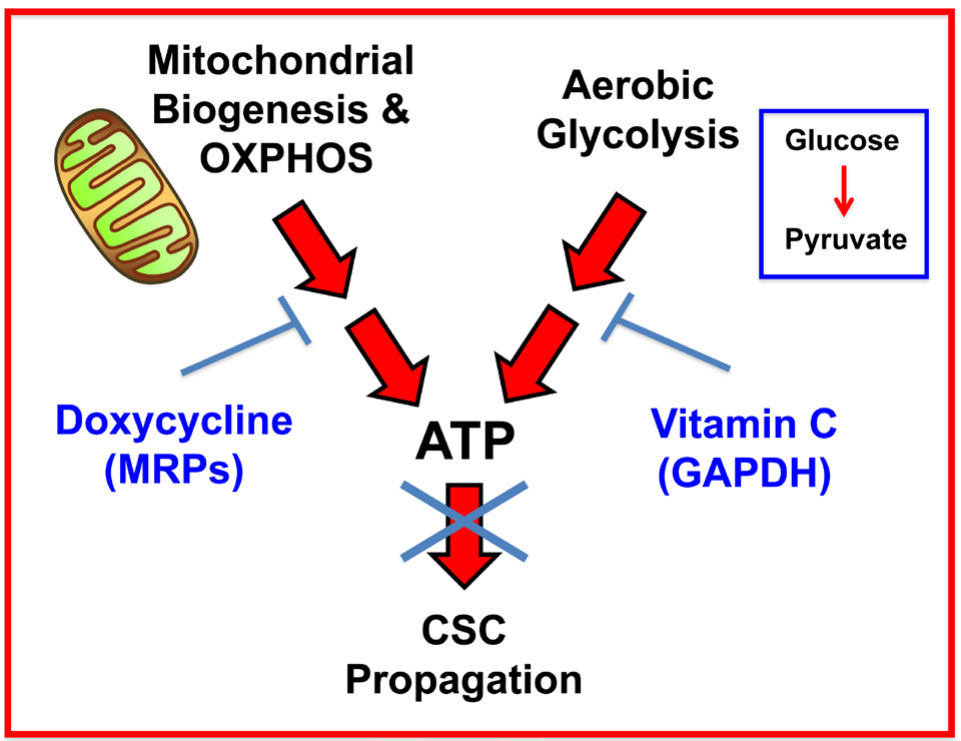
Vitamin C and Doxycycline: A synthetic lethal combination therapy for eradicating CSCs Note that both OXPHOS and the glycolytic pathway jointly contribute to ATP production. Doxycycline inhibits mitochondrial biogenesis and OXPHOS, by acting *via* mitochondrial ribosomal proteins (MRPs); Vitamin C inhibits glycolytic metabolism by targeting and inhibiting the enzyme GAPDH. Therefore, their use together, as a sequential drug combination, will more severely target cell metabolism and energy production, thereby preventing or blocking the propagation of CSCs.

Our new therapeutic strategy should provide for the more efficient eradication of CSCs using Doxycycline, as well as a practical means for solving the potential problem of Doxycycline resistance in CSCs. As such, we suggest a new synthetic lethal strategy for eradicating CSCs, by employing i) Doxycycline (to target mitochondria) and ii) Vitamin C (to target glycolysis) (Figure 12). Use of this combined metabolic strategy should help prevent CSCs from exploiting the essential nutrients that they normally derive from the tumor microenvironment.

### Chronic Doxycycline treatment functionally confers a mitochondrial-deficient metabolic phenotype, actively suppressing CSC activity

Previous studies have shown that human cancer cells lacking mt-DNA [called rho(0) cells] have largely lost their ability to undergo mitochondrial OXPHOS and they fail to initiate tumors *in vivo*, as determined by using pre-clinical animal models to assess tumorigenicity [3]. Importantly, their ability to undergo OXPHOS and to form tumors was effectively restored by genetic replacement of their mt-DNA [5]. As such, it appears that mitochondrial oxidative function and mt-DNA are required for energetically initiating the process of tumorigenesis, across multiple cancer types [3, 5].

Here, we observed that MCF7-DoxyR cells strongly phenocopy the metabolic behavior of mt-DNA deficient (rho(0)) cells, by exhibiting a purely glycolytic phenotype. Consistent with this hypothesis, MCF7-DoxyR cells lack the expression of four mitochondrial proteins normally encoded by mt-DNA (MT-ND3, MT-CO2, MT-ATP6 and MT-ATP8) and they exhibit a near complete loss of OXPHOS activity, with a strong induction of aerobic glycolysis. MCF7-DoxyR cells also show significant functional reductions in cell proliferation and migration, as well as a loss of CSC propagation - a surrogate marker of tumor-initiating activity.

Therefore, chronic doxycycline treatment provides a pharmacological means to mimic a rho(0) cell phenotype, to therapeutically reduce tumor growth and to avoid tumor recurrence. However, Doxycycline-resistance still remains a valid concern.

### Overcoming resistance to Doxycycline in cancer cells, using metabolic inflexibility, synthetic lethality and Vitamin C

Resistance to anti-cancer therapy remains as one of the key issues in cancer patient management. Treatment failure is regarded as an alarming outcome of numerous different therapeutic approaches. Indeed, the use of combination strategies aimed at hitting multiple aspects of tumor progression is currently considered as a promising tool to overcome resistance. Mounting evidence suggests that CSCs act as the main promoter of tumor recurrence and patient relapse [1–5]. Thus, a better understanding of the biological and biochemical behavior of CSCs during drug resistance may unveil new vulnerabilities, to be exploited in a therapeutic setting.

In this context, our data indicates that a metabolic shift from oxidative to glycolytic metabolism represents an escape mechanism for breast cancer cells chronically-treated with a mitochondrial stressor like Doxycycline, as mitochondrial dys-function leads to a stronger dependence on glucose.

Our current findings are in line with previous studies showing the highly plastic nature of CSCs allows them to adjust and adapt their metabolic environment, in order to maintain their distinctive properties, in a hostile tumor microenvironment, often characterized by an inadequate nutrient and oxygen supply (reviewed in [23]). Here, we have taken advantage of the glycolytic shift exhibited by DoxyR CSCs, as we have used several glycolysis inhibitors with the aim to turn their strict metabolic inflexibility, into a lethal phenotype. Among the agents tested in the our study, Vitamin C has been demonstrated to selectively kill cancer cells *in vitro* and to inhibit tumor growth in experimental mouse models [24, 25]. Remarkably, many of these actions have been attributed to the ability of Vitamin C to act as a glycolysis inhibitor, by targeting GAPDH and depleting the NAD pool [22, 26, 27].

In this context, we have previously demonstrated that Vitamin C effectively inhibits 3D breast tumor spheroid formation, with an IC-50 of 1 mM, suggesting that this micronutrient also works as an inhibitor of CSCs [13], whose activity is critically dependent on an active mitochondrial TCA cycle and OXPHOS. In contrast, here we show that DoxyR CSCs are more vulnerable to the inhibitory effects of Vitamin C, at 4- to 10-fold lower concentrations, between 100 to 250 μM. These findings are further supported by clinical studies showing that the concurrent use of Vitamin C, with standard chemotherapy, reduces tumor recurrence and patient mortality [28, 29].

It is worth noting that Vitamin C plasma levels vary considerably with the route of administration. For instance, pharmacokinetic studies performed by different research groups have assessed that, after oral administration, Vitamin C plasma levels reach concentrations of ∼70-220 μM [reviewed in reference [30], which represents the maximum tolerated oral dose. By contrast, Padayatty and co-workers found that, compared to oral intake, intravenous administration results in 30- to 70- fold higher plasma concentrations of Vitamin C [31]. Furthermore, consumption of 5 to 9 servings of fruits and vegetables per day allows plasma levels of Vitamin C to reach up to 80 μM at steady-state, with peak values of 220 μM [31]. Remarkably, an intravenous infusion of Vitamin C can reach plasma levels of 15,000 μM (i.e., 15 mM). Interestingly, doses of up to 50 grams per day, infused slowly, didn't exhibit any toxic side effects on cancer patients [30]. These observations suggest that intravenous administration of Vitamin C may have a role in cancer treatment, as this route allows higher plasma concentrations than those achievable with the maximum tolerated oral dose.

Previous studies have demonstrated that Vitamin C behaves as a potent dietary antioxidant, as well as a pro-oxidant. This pro-oxidant activity results from Vitamin C's action on metal ions, which generates free radicals and hydrogen peroxide, and is associated with cell toxicity. Of note, it has been shown that high-dose Vitamin C is more cytotoxic to cancer cells than to normal cells [32, 33]. This selectivity appears to be due to the higher catalase content observed in normal cells (∼10-100 fold greater), as compared to tumor cells. Hence, Vitamin C may be regarded as a safe agent that selectively targets cancer cells.

A recent study performed on a panel of cancer cells (A431, Panc-1, HeLa, HT29, and MCF7) showed that Vitamin C only affects cell viability at concentration of ∼3 to 10 mM [34], thus providing more evidence to support a lack of toxicity for low micromolar concentrations of Vitamin C.

Similarly, phase I and II clinical trials, designed to deliver high-dose intravenous Vitamin C, have shown a lack of toxic side effects for concentrations of up to 292 μM [35]. Taken together with these findings, our data suggest that Vitamin C's action as a glycolytic inhibitor may represent a safe and effective strategy to be used in combination therapies, with conventional anticancer drugs, as well as with Doxycycline.

Because of our success with 2-DG and Vitamin C, we explored additional FDA-approved drugs with glycolysis-inhibiting activity that could be repurposed to eradicate CSC propagation, in combination with Doxycycline. For example, we demonstrated that the LDH enzyme inhibitor Stiripentol is also effective at targeting DoxyR CSCs; this drug is currently used clinically as an anti-epileptic in children.

We also further explored other suitable metabolic approaches to overcome Doxycycline resistance. In this context, we evaluated the efficacy of a panel of compounds that share the ability to impair mitochondrial function (OXPHOS), as a common off-target side-effect. This approach was based on the assumption that DoxyR cells, which exhibit altered oxidative metabolism, are extremely sensitive to the induction of additional mitochondrial dysfunction. These effective compounds included four FDA-approved drugs, such as Atovaquone, Irinotecan, Sorafenib, and Niclosamide, as well as the natural product Berberine.

Interestingly, we observed that Chloroquine also reduces the spheroid forming efficiency of DoxyR cells, indicating that inhibition of autophagy may represent another effective combination strategy, to sensitize breast cancer cells to the actions of Doxycycline.

We believe that the emerging functional relationship between metabolism and stemness, also known as “metabo-stemness” [36], holds great promise for the future of anti-cancer therapy. Thus, our novel findings may pave the way for the discovery and validation of more effective therapeutic strategies to fully eradicate CSCs, ultimately preventing treatment failure and minimizing metastatic dissemination.

### Synergistic effects of Doxycycline and Vitamin C in the treatment of infectious disease states

Is there any precedent for the use of Doxycycline in combination with Vitamin C, in clinical trials? Interestingly, another group published a report on a randomized clinical trial of the effects of Vitamin C on dyspareunia and vaginal discharge, in women receiving Doxycycline and Triple sulfa for chlamydial cervicitis infections [37]. Importantly, they concluded that the cure rate was nearly 5-fold higher in the patients that received Vitamin C, together with antibiotic therapy [37]. So, the concurrent use of Doxycycline and Vitamin C, in the context of this infectious disease, appeared to be highly synergistic in patients.

Similarly, Goc et al., 2016, showed that Doxycycline is synergistic *in vitro* with certain phytochemicals and micronutrients, including Vitamin C, in the *in vitro* killing of the vegetative spirochete form of Borrelia spp., the causative agent underlying Lyme disease [38]. Vitamin C has also been shown to be synergistic with Tetracycline and Chloramphenicol, against the pathogenic bacteria, Pseudomonas aeruginosa [39]. However, in the above examples, no follow-up mechanistic studies were conducted to determine exactly why Doxycycline, Tetracycline and Vitamin C were somehow synergistic.

## CONCLUSIONS

Numerous functional studies have now directly shown that mitochondria are an important new therapeutic target in cancer cells [3, 5, 8–21, 40–53]. Since Doxycycline, an FDA-approved antibiotic, behaves as an inhibitor of mitochondrial protein translation, it may have therapeutic value in the specific targeting of mitochondria in cancer cells. However, in this paper, we have identified a novel metabolic mechanism by which CSCs successfully escape from the anti-mitochondrial effects of Doxycycline, by assuming a purely glycolytic phenotype. Therefore, DoxyR CSCs are then more susceptible to other metabolic perturbations, because of their metabolic inflexibility, allowing for their eradication with natural products and other FDA-approved drugs. Thus, understanding the metabolic basis of Doxycycline-resistance has ultimately helped us to develop a new synthetic lethal strategy, for more effectively targeting CSCs.

## MATERIALS AND METHODS

### Materials

Doxycycline, Ascorbic Acid, 2-Deoxy-D-glucose (2-DG), Irinotecan, Berberine Chloride, Niclosamide, Chloroquine diphosphate, Stiripentol and Atovaquone were all purchased from Sigma Aldrich. Sorafenib was obtained from Generon. All compounds were dissolved in DMSO, except Ascorbic Acid, 2-deoxy-D-glucose (2-DG) and Chloroquine diphosphate, which were dissolved in cell culture medium.

### Cell cultures

MCF7 breast cancer cells were obtained from ATCC and cultured in DMEM (Sigma Aldrich). MCF-7 cells resistant to Doxycycline (MCF7 DoxyR) were selected by a stepwise exposure to increasing concentration of Doxycycline. In particular, wild type MCF7 cells were initially exposed to 12.5 μM Doxycycline and the dose gradually increased to 50 μM over a 3-month period. The population of resistant cells, named MCF7 DoxyR, was selected after 3 weeks of treatment with 12.5 μM Doxycycline, followed by 3 weeks of treatment with 25 μM Doxycycline. MCF7 DoxyR cells were routinely maintained in regular medium supplemented with 25 μM Doxycycline.

### Mammosphere formation

A single cell suspension of MCF7 or MCF7 DoxyR cells was prepared using enzymatic (1x Trypsin-EDTA, Sigma Aldrich), and manual disaggregation (25 gauge needle) [54]. Cells were then plated at a density of 500 cells/cm^2^ in mammosphere medium (DMEM-F12/B27/20-ng/ml EGF/PenStrep) in nonadherent conditions, in culture dishes coated with (2-hydroxyethylmethacrylate) (poly-HEMA, Sigma), in the presence of treatments, were required. Cells were grown for 5 days and maintained in a humidified incubator at 37°C at an atmospheric pressure in 5% (v/v) carbon dioxide/air. After 5 days for culture, spheres > 50 μm were counted using an eye piece graticule, and the percentage of cells plated which formed spheres was calculated and is referred to as percentage mammosphere formation. Mammosphere assays were performed in triplicate and repeated three times independently.

### Evaluation of mitochondrial mass and function

To measure mitochondrial mass by FACS analysis, cells were stained with MitoTracker Deep Red (Life Technologies), which localizes to mitochondria regardless of mitochondrial membrane potential. Cells were incubated with pre-warmed MitoTracker staining solution (diluted in PBS/CM to a final concentration of 10 nM) for 30-60 min at 37°C. All subsequent steps were performed in the dark. Cells were washed in PBS, harvested, re-suspended in 300 μL of PBS and then analyzed by flow cytometry (Fortessa, BD Bioscience). Data analysis was performed using FlowJo software. Extracellular acidification rates (ECAR) and real-time oxygen consumption rates (OCR) for MCF7 cells were determined using the Seahorse Extracellular Flux (XFe-96) analyzer (Seahorse Bioscience) [15]. Briefly, 15,000 MCF7 and MCF7 DoxyR cells per well were seeded into XFe-96 well cell culture plates for 24h. Then, cells were washed in pre-warmed XF assay media (or for OCR measurement, XF assay media supplemented with 10mM glucose, 1mM Pyruvate, 2mM L-glutamine and adjusted at 7.4 pH). Cells were then maintained in 175 μL/well of XF assay media at 37C, in a non-CO_2_ incubator for 1 hour. During the incubation time, 5 μL of 80mM glucose, 9 μM oligomycin, and 1 M 2-deoxyglucose (for ECAR measurement) or 10μM oligomycin, 9 μM FCCP, 10 μM Rotenone, 10 μM antimycin A (for OCR measurement), were loaded in XF assay media into the injection ports in the XFe-96 sensor cartridge. Data set was analyzed by XFe-96 software after the measurements were normalized by protein content (SRB). All experiments were performed three times independently.

### ALDEFLUOR assay and separation of the ALDH positive population

ALDH activity was assessed by FACS analysis (Fortessa, BD Bioscence) in MCF7 cells and MCF7 DoxyR cells. The ALDEFLUOR kit (StemCell Technologies) was used to isolate the population with high ALDH enzymatic activity. Briefly, 1 × 10^5^ MCF7 and MCF7 DoxyR cells were incubated in 1ml ALDEFLUOR assay buffer containing ALDH substrate (5 μl/ml) for 40 minutes at 37°C. In each experiment, a sample of cells was stained under identical conditions with 30 μM of diethylaminobenzaldehyde (DEAB), a specific ALDH inhibitor, as a negative control. The ALDEFLUOR-positive population was established in according to the manufacturer's instructions and was evaluated in 3 × 10^4^ cells. Data analysis was performed using FlowJo software.

### Anoikis assay

MCF7 and MCF7 DoxyR cells were seeded on low-attachment plates to enrich for the CSC population [54]. Under these conditions, the non-CSC population undergoes anoikis (a form of apoptosis induced by a lack of cell-substrate attachment) and CSCs are believed to survive. The surviving CSC fraction was analyzed by FACS analysis. Briefly, 1 × 10^5^ MCF7 and MCF7 DoxyR monolayer cells were seeded for 48h in 6-well plates. Then, cells were trypsinized and seeded in low-attachment plates in mammosphere media. After 10h, cells were spun down and incubated with CD24 (IOTest CD24-PE, Beckman Coulter) and CD44 (APC mouse Anti-Human CD44, BD Pharmingen) antibodies for 15 minutes on ice. Cells were rinsed twice and incubated with LIVE/DEAD dye (Fixable Dead Violet reactive dye; Life Technologies) for 10 minutes. Samples were then analyzed by FACS (Fortessa, BD Bioscence). Only the live population, as identified by the LIVE/DEAD dye staining, was analyzed for CD24/CD44 expression. Data were analyzed using FlowJo software.

### Label-free semi-quantitative proteomics analysis

Cell lysates were prepared for trypsin digestion by sequential reduction of disulphide bonds with TCEP and alkylation with MMTS. Then, the peptides were extracted and prepared for LC-MS/MS. All LC-MS/MS analyses were performed on an LTQ Orbitrap XL mass spectrometer (Thermo Scientific, San Jose, CA) coupled to an Ultimate 3000 RSLC nano system (Thermo Scientific, formerly Dionex, The Netherlands). Xcalibur raw data files acquired on the LTQ-Orbitrap XL were directly imported into Progenesis LCMS software (Waters Corp., Milford, MA, formerly Non-linear dynamics, Newcastle upon Tyne, UK) for peak detection and alignment. Data were analyzed using the Mascot search engine. Five technical replicates were analyzed for each sample type [8, 12].

### Immuno-blot analysis

MCF7 and MCF7 DoxyR cells protein lysates were electrophoresed through a reducing SDS/10% (w/v) polyacrylamide gel, electroblotted onto a nitrocellulose membrane and probed with primary antibodies against phosphorylated AKT (Ser 473) and ATK (Cell Signaling), Phopshorylated ERK 1/2 (E-4), ERK2 (C-14), TOMM20 (F-10) and β-actin (C2), all purchased from Santa Cruz Biotechnology. Proteins were detected by horseradish peroxidase-linked secondary antibodies and revealed using the SuperSignal west pico chemiluminescent substrate (Fisher Scientific).

### Click-iT EdU proliferation assay

48h after seeding MCF7 and MCF7 DoxyR were subjected to proliferation assay using Click-iT Plus EdU Pacific Blue Flow Cytometry Assay Kit (Life Technologies), customized for flow cytometry. Briefly, cells were treated with 10 μM EdU for 2 hours and then fixed and permeabilized. EdU was detected after permeabilization by staining cells with Click-iT Plus reaction cocktail containing the Fluorescent dye picolylazide for 30 min at RT. Samples were then washed and analyzed using flow cytometer (Fortessa, BD Bioscence). Background values were estimated by measuring non-EdU labeled, but Click-iT stained cells. Data were analyzed using FlowJo software.

### Migration assay

MCF7 and MCF7 DoxyR cells were allowed to grow in regular growth medium until they were 70-80 % confluent. Next, to create a scratch of the cell monolayer, a p200 pipette tip was used. Cells were washed twice with PBS and then incubated at 37°C in regular medium for 24h. The migration assay was evaluated using Incucyte Zoom (Essen Bioscience) [55]. The rate of migration was measured by quantifying the % of wound closure area, determined using the software ImageJ, according to the formula:

% of wound closure = [(At = 0 h - At = Δ h)/At = 0 h] × 100%

### Statistical analysis

Data is represented as the mean ± standard error of the mean (SEM), taken over ≥ 3 independent experiments, with ≥ 3 technical replicates per experiment, unless otherwise stated. Statistical significance was measured using the *t*-test. P ≤ 0.05 was considered significant.
